# Antioxidant Activity of Stallion Spermatozoa After Cryopreservation with Natural Antioxidant-Supplemented Extenders

**DOI:** 10.3390/ani16111704

**Published:** 2026-06-02

**Authors:** Stefano Cecchini Gualandi, Alessandro Pistone, Angela Ostuni, Graziano Preziosi, Maria Antonietta Ferrara, Raffaele Boni

**Affiliations:** 1 Department of Basic and Applied Sciences, University of Basilicata, Via dell’Ateneo Lucano, 10, 85100 Potenza, Italy; alessandro.pistone@unibas.it (A.P.); angela.ostuni@unibas.it (A.O.); 2 Institute of Applied Sciences and Intelligent Systems (ISASI), Unit of Naples, Italian National Research Council (CNR), Via Pietro Castellino 111, 80131 Napoli, Italy; graziano.preziosi@na.isasi.cnr.it (G.P.); antonella.ferrara@na.isasi.cnr.it (M.A.F.)

**Keywords:** antioxidant activity, equine reproduction, freezing extender, natural antioxidant, oxidative stress, semen cryopreservation, sperm kinetics

## Abstract

Cryopreservation of stallion semen is associated with oxidative stress (OS), which can impair sperm function and fertility. This study evaluated antioxidant activities in seminal plasma and sperm cytosols and investigated their relationships with selected sperm functional parameters following cryopreservation, with or without antioxidant supplementation. Semen was collected from ten fertile stallions and processed using a split-ejaculate design, including fresh semen and six freezing treatments: HF-20 extender alone; HF-20 supplemented with matcha, spirulina, horseradish, or quercetin; and a commercial extender (INRA Freeze). Total antioxidant capacity (FRAP) and enzymatic activities (superoxide dismutase, SOD; catalase, CAT; and glutathione reductase, GR) were measured in seminal plasma and sperm lysates. Linear regression analyses revealed significant associations between seminal plasma and fresh spermatozoa with respect to SOD and GR activities. In frozen-thawed semen, FRAP and CAT activities differed between samples cryopreserved with and without antioxidant supplementation. Significant correlations were observed among antioxidant activities, sperm kinetics, OS markers, and DNA fragmentation indices. Principal component analysis provided an exploratory overview of multidimensional patterns of covariation among sperm kinetics, redox balance, and nuclear fragmentation, explaining for 73% of the total variance. Overall, the results suggest complex associations between the antioxidant system and sperm quality and indicate that antioxidant supplementation of freezing extenders may modulate the redox status of stallion sperm after thawing.

## Simple Summary

Semen cryopreservation is widely used in horse breeding; however, the freezing-thawing process can impair sperm function, primarily due to oxidative stress. Antioxidants may help protect sperm cells during this process. In this study, we evaluated the antioxidant potential of stallion sperm after freezing using a semi-defined extender (HF-20) either supplemented with natural antioxidants (matcha, spirulina, horseradish, and quercetin) or not, and compared the results with those obtained using a commercial extender as a benchmark. We measured antioxidant activity and investigated its relationships with selected sperm functional variables, including sperm kinetics, energy metabolism, oxidative/nitrosative markers, and DNA integrity. Although no significant improvement in sperm functionality was observed following supplementation of the freezing extender with the tested antioxidants, the antioxidants exhibited specific effects: quercetin increased total non-enzymatic antioxidant power, while matcha enhanced catalase activity. Correlation and multivariate analyses revealed clear associations between antioxidant defenses, sperm function, and oxidative damage. Overall, these findings suggest that natural antioxidant extracts added to the freezing extender do not produce a substantial improvement in sperm function, but they may influence the antioxidant capacity of spermatozoa subjected to cryopreservation. These findings provide a useful contribution toward improving cryopreservation strategies in equine reproduction.

## 1. Introduction

Semen cryopreservation is a key technology in equine reproduction, enabling long-term storage of genetic material and supporting breeding management and genetic dissemination [1,2]. However, the efficiency of this technique in stallions remains highly variable, with inconsistent post-thaw sperm quality among individuals and ejaculates [3]. This variability is largely attributed to differences in intrinsic sperm cryotolerance, which cannot be easily predicted or standardized, and represents a major limitation in equine reproductive biotechnology [4,5].

Among the variables involved in cryodamage, oxidative stress (OS) plays a pivotal role [6]. During semen processing and cryopreservation, sperm cells are exposed to increased levels of reactive oxygen and nitrogen species (ROS and RNS), resulting from both endogenous metabolism and external stressors [7,8]. Stallion spermatozoa are particularly susceptible to oxidative damage due to their use of mitochondrial oxidative phosphorylation for energy production and their limited cytoplasmic antioxidant capacity [9,10,11]. Furthermore, the dilution or removal of seminal plasma (SP) during semen handling reduces the availability of endogenous antioxidant systems, thereby further exacerbating redox imbalance in spermatozoa [12,13].

Notably, reactive species are not exclusively detrimental to sperm function. As highlighted by O’Flaherty et al. [14], ROS are involved in key physiological processes, including capacitation and intracellular signaling, when maintained within controlled levels. Therefore, sperm functionality depends on a finely regulated redox balance rather than the mere absence of oxidative molecules. However, cryopreservation disrupts this equilibrium, leading to excessive accumulation of reactive species, which in turn impairs sperm function.

The detrimental effects of OS during cryopreservation are multifactorial. Lipid peroxidation compromises membrane integrity and fluidity, affecting sperm motility and viability [15,16]. Oxidative damage to proteins alters the activity and structural stability of antioxidant enzymes, while DNA fragmentation negatively impacts fertilization potential and embryo development [17]. In addition, cryopreservation induces mitochondrial dysfunction and capacitation-like changes, further reducing sperm lifespan and functionality after thawing [18]. These alterations collectively contribute to the decline in sperm quality typically observed following freezing-thawing procedures.

A major challenge in equine semen cryopreservation is the strong inter- and intra-variability observed in stallions; factors such as age, metabolic status, and baseline sperm quality significantly affect cryotolerance even in sperm samples collected from the same stallion [5,13,19,20,21]. In addition, variability in the endogenous antioxidant capacity of sperm and SP contributes to differences in susceptibility to OS, further complicating the interpretation of treatment effects [22,23]. These aspects highlight the complexity of sperm cryobiology and the need for integrative approaches capable of capturing the multidimensional nature of sperm function.

Given the central role of OS, considerable efforts have been directed toward improving cryopreservation outcomes through the supplementation of semen extenders with antioxidant compounds. In recent years, natural antioxidants have attracted increasing attention due to their complex composition and the potential synergistic effects of multiple antioxidant activities. Plant extracts or plant-derived compounds rich in polyphenols, flavonoids, and carotenoids, as well as microalgae such as *Spirulina platensis*, have been proposed either as feed supplements [24,25,26] or additives in extenders for both chilled and cryopreserved semen [15,27,28]. These compounds can exert multiple biological effects, including free radical scavenging, modulation of cellular signaling pathways, and protection of sperm membrane structures [29,30]. Nevertheless, the results reported in the literature are variable, likely reflecting differences in experimental conditions, antioxidant type and concentration, and intrinsic sperm characteristics. Thus, the effectiveness of antioxidant supplementation in stallion semen remains inconsistent, suggesting that a simple reduction in ROS levels may not be sufficient to assure a high sperm quality and maintain an optimal redox balance.

Based on these considerations, we developed a comprehensive study aimed at evaluating the potential benefits on stallion sperm quality resulting from the addition of natural antioxidant extracts to the freezing extender. Specifically, matcha, spirulina, and horseradish extracts, or purified quercetin were added into a semi-defined freezing extender (HF-20). A commercial extender (INRA Freeze^®^) was also included in this study and used as benchmark. This study considered numerous aspects associated with the quality of frozen semen in the presence of these treatments. In the present report, attention was focused on the antioxidant potential, evaluated as total antioxidant activity by FRAP assay, as well as through the analysis of individual enzymatic activities of superoxide dismutase (SOD), catalase (CAT), and glutathione reductase (GR) in seminal plasma and in sperm lysates, in both fresh and thawed samples frozen with or without antioxidant supplementation. These results were integrated with the findings on sperm functionality previously reported in an earlier paper and were analyzed through correlation and principal component analyses.

## 2. Materials and Methods

### 2.1. Materials

All chemicals used for the analyses, unless otherwise specified, were supplied by Sigma-Aldrich (Milan, Italy).

### 2.2. Sample Collection and Preparation

Between February and May 2025, a single ejaculate was collected from ten clinically healthy, proven fertile Salernitano stallions (4–17 years old). The animals were housed individually in paddocks at the Regional Center for Equine Genetic Improvement (Caserta, Italy), where well-established and appropriate management and environmental conditions were maintained. Throughout the study period, stallions received a conventional feeding regimen consisting of hay and a commercial concentrate, had unrestricted access to water, and were not provided with any antioxidant dietary supplementation. In this Center, all the operations of semen collection were carried out.

The Regional Center for Equine Genetic Improvement is a facility officially accredited by the Campania Regional Authority (authorization no. U1500083 CE000642004). It operates in accordance with current regulations concerning health status, biosecurity measures, and animal welfare. All experimental procedures complied with the provisions of European Directive 2010/63/EU and the related Italian legislation (D. Lgs. 26/2014), ensuring particular attention to the reduction in animal stress and to limiting the number of animals involved in the study.

For each stallion, part of the collected ejaculate was rapidly brought to 4 °C to be processed upon arrival at the Laboratory of Animal Reproductive Biology and Technology approximately two hours after collection. Seminal plasma (SP) was obtained through two sequential centrifugation steps (400× *g* for 10 min at 4 °C followed by 1500× *g* for 10 min at 4 °C) to remove sperm cells and residual debris. The recovered SP was then stored at −80 °C until further analyses.

The remaining semen was diluted with INRA 96^®^ for shipping and subjected to a gradual decrease in temperature to approximately 20 °C [15]. In the analysis laboratory, the semen of each stallion was centrifuged (400× *g* for 10 min). Each pellet was subdivided into seven aliquots, yielding one fresh sample (resuspended in INRA 96) and six samples intended for different cryopreservation treatments. For each donor, the following experimental groups were established: HF-20 alone or supplemented with 10 µg mL^−1^ matcha, 5 µg mL^−1^ spirulina, 5 µg mL^−1^ horseradish extracts, or 5 µg mL^−1^ quercetin (Q4951, Sigma-Aldrich, Milan, Italy). These dosages were selected based on a preliminary evaluation aimed at identifying the most appropriate concentrations and were reported in an earlier study [15]. The same study also reported the results of a series of analytical assays, including total antioxidant capacity (TAC), total reducing power (TRP), free radical scavenging activity (FRSA), total polyphenol content (TPC), and total flavonoid content (TFC), aimed at evaluating the antioxidant properties and the polyphenol and flavonoid contents of these extract powders. All extract powders were pre-diluted in PBS (1 mg mL^−1^), whereas quercetin was pre-diluted in DMSO (1 mg mL^−1^) before addition to the freezing extender. An additional aliquot of spermatozoa was diluted in INRA-Freeze. Detailed protocols for semen processing, preparation of antioxidant extracts and evaluation of their antioxidant potential, freezing procedures, and post-thaw handling are reported in [15].

### 2.3. Sperm Kinetics, Bioenergetics, Oxidative/Nitrosative Stress Markers and DNA Fragmentation Index

Sperm motility was analyzed using a computer-assisted sperm analysis system (SCA 5.0; Microptic, Barcelona, Spain) according to the methodology previously described by [15]. The motility parameters considered included total motility (TM), progressive motility (PM), curvilinear velocity (VCL), straight-line velocity (VSL), and average path velocity (VAP).

Indicators of mitochondrial activity and sperm oxidative/nitrosative stress markers were also evaluated. Specifically, mitochondrial membrane potential (MMP), lipid peroxidation (LPO), intracellular reactive oxygen species (ROS), and nitric oxide (NO) production were determined using the fluorescent probes JC-1, C11-BODIPY^581/591^, H_2_DCFDA, and DAF-FM diacetate (Thermo Fisher Scientific, Waltham, MA, USA), respectively, following previously established procedures [15]. Fluorescence signals were quantified by fluorescence spectroscopy.

Sperm DNA integrity was assessed by calculating the DNA Fragmentation Index (DFI) through two complementary techniques: a direct assay, the terminal deoxynucleotidyl transferase-mediated dUTP nick-end labelling test (APO-BrdU TUNEL), and an indirect method, the sperm chromatin structure assay (SCSA) [15]. The TUNEL assay detects DNA strand breaks by labelling free 3′-OH terminal with the thymidine analogue 5-bromo-2′-deoxyuridine-5′-triphosphate (BrdUTP) through the action of terminal deoxynucleotidyl transferase. Instead, SCSA evaluates the susceptibility of sperm chromatin to acid-induced denaturation and exploits the metachromatic properties of acridine orange (AO) to differentiate intact double-stranded DNA from denatured single-stranded DNA. A detailed description of these analytical procedures and the corresponding findings has been reported previously [15].

### 2.4. Obtaining Sperm Lysate and Evaluation of Antioxidant Activities

Fresh and frozen-thawed sperm (approximately 1 × 10^7^ per sample) from different experimental groups were washed in PBS twice by centrifugation at 400× *g* for 10 min at 4 °C, and the pellet was stored at −80 °C. Subsequently, each frozen sample was thawed and supplemented with 250 µL RIPA buffer (0.1% sodium dodecyl sulfate, 1% NP-40, 0.5% sodium deoxycholate in PBS, pH 7.4) enriched with a protease and phosphatase inhibitors (Roche Diagnostics, Mannheim, Germany), and lysed by sonication (30 s at 37% power on ice) using a Bandelin Sonoplus HD2070 (Bandelin electronic GmbH & Co. KG, Berlin, Germany). The lysates were centrifuged at 10,000× *g* for 10 min at 4 °C, and protein concentration was measured by the Bradford method using a total protein reagent (cod. B6916) and bovine serum albumin (BSA) as standard.

Non-enzymatic antioxidant capacity was evaluated using the ferric reducing antioxidant power (FRAP) assay, as described by [31]. Absorbance was recorded at 593 nm using a microplate reader (Model 550, Bio-Rad Laboratories, Segrate, Milan, Italy). A calibration curve was prepared using iron(II) sulfate heptahydrate (FeSO_4_·7H_2_O). Antioxidant capacity was expressed as µM FeSO_4_·7H_2_O equivalents and normalized to mg^−1^ protein content.

Enzymatic antioxidant activities (superoxide dismutase, SOD; catalase, CAT and glutathione reductase, GR) were determined using assay kits (SOD cod. 19160; CAT cod. 219265, GR cod. GRSA) (Sigma-Aldrich, St. Louis, MO, USA). Each sample was analyzed in duplicate, and the mean value was entered into the datasheet. SOD activity was measured based on the inhibition of WST-1 reduction by superoxide anions generated through the xanthine/xanthine oxidase system, with decreased formazan formation proportional to enzyme activity. CAT activity was determined by measuring formaldehyde production from the reaction of methanol with hydrogen peroxide (H_2_O_2_) in the presence of catalase; formaldehyde was detected using Purpald, which forms a purple chromophore upon oxidation. GR activity was assessed by monitoring the reduction of oxidized glutathione (GSSG) to reduced glutathione (GSH) in the presence of NADPH. The GSH produced subsequently reacts with DTNB to form TNB, and the resulting increase in absorbance is proportional to the enzyme activity. Enzymatic activities were expressed as inhibition rate (%), nM min^−1^ mL^−1^ and mU mL^−1^, respectively, normalized to mg^−1^ protein content.

### 2.5. Statistical Analysis

Unless otherwise specified, the statistical analyses were performed using the open-source Jamovi software (Jamovi project, 2026, version 2.7.18).

To explore the relationship between antioxidant activities (FRAP, SOD, CAT, and GR) in SP and the corresponding activities in fresh semen, linear regression analyses were performed. Statistical significance was set at *p* < 0.05.

Antioxidant activities of frozen–thawed semen samples were analyzed using linear mixed models (LMMs) to evaluate the effect of different freezing treatments under a split-ejaculate design. In this model, freezing treatment was included as a fixed effect, while stallion was specified as a random intercept. Degrees of freedom were estimated using the Satterthwaite approximation. When significant effects were detected, post hoc pairwise comparisons among treatments were performed using estimated marginal means with Tukey’s adjustment for multiple testing. Model assumptions were evaluated by visual inspection of residual plots to assess normality and homoscedasticity. Statistical significance was set at *p* < 0.05.

Pearson’s correlation coefficients were calculated to assess associations among variables related to antioxidant activities, sperm kinetics, bioenergetics, oxidative/nitrosative stress markers, and DNA fragmentation indices. The resulting correlation matrix was visualized as a heatmap using a diverging color scale centered at zero and fixed to the theoretical limits of Pearson’s r (−1 to +1).

Additionally, principal component analysis (PCA) was conducted on standardized variables using an oblique Promax rotation to identify latent multidimensional structures within sperm kinematic, OS, antioxidant, and DNA fragmentation parameters; component retention was determined based on eigenvalues > 1, scree plot inspection, and parallel analysis, after verifying sampling adequacy by Kaiser–Meyer–Olkin (KMO) test and sphericity by Bartlett’s test. PCA was performed using the open-source JASP (JASP team, 2025, version 0.95.4).

## 3. Results

### 3.1. Antioxidant Activity in Seminal Plasma and Fresh Sperm Lysates

Table 1 presents the mean activities of selected antioxidant variables measured in SP and sperm lysates from fresh samples. Substantial differences can be observed between these measurements, with values that are twofold or fivefold higher in the case of FRAP and antioxidant enzymatic activities, respectively, in the sperm cytosol compared with those in the seminal plasma (SP). However, within each matrix, the percentage differences among the individual enzymatic activities remained of similar magnitude.

**Figure 1:**
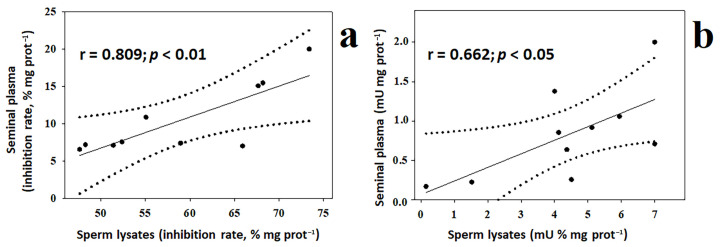
Regression lines and correlation coefficients (r) of enzymatic antioxidant activities between fresh sperm lysates and seminal plasma. (**a**) SOD activity; (**b**) GR activity. The dashed lines represent the 95% confidence interval curves.

Regression analysis comparing antioxidant activities in SP with the corresponding activities measured in sperm lysates from fresh samples revealed significant correlations for SOD (r = 0.809; *p* < 0.01) and GR (r = 0.662; *p* < 0.05) activities (Figure 1). In contrast, no significant association was observed for FRAP (*p* = 0.263) or CAT activity (*p* = 0.200).

### 3.2. Selected Parameters Related to Sperm Quality

Table 2 reports the results of sperm kinetics, mitochondrial activity, oxidative/nitrosative stress markers, and DNA fragmentation indices in fresh stallion spermatozoa (T0) and in samples frozen with or without natural extracts with antioxidant activity, quercetin, or the commercial freezing extender INRA Freeze.

Data analysis showed that all sperm kinematic parameters and DNA fragmentation indices were significantly different (*p* < 0.01) between fresh and frozen-thawed spermatozoa, both in the absence and in the presence of antioxidant supplementation. In contrast, no differences were observed between fresh and frozen spermatozoa with respect to mitochondrial activity and oxidative/nitrosative stress markers. Analysis restricted to the frozen-thawed sperm groups did not reveal any significant effects associated with antioxidant supplementation, nor were any differences observed compared with spermatozoa frozen using the reference commercial extender. This may be attributed to the strong individual effect (*p* < 0.01) detected for all sperm kinematic parameters and DFI^SCSA^. Metabolic parameters related to MMP, lipid peroxidation, and oxidative/nitrosative stress markers also showed significant individual variability (*p* < 0.05), whereas this variability was less pronounced in the DFI^TUNEL^.

### 3.3. Antioxidant Activity in Frozen-Thawed Sperm Cells Cryopreserved with Different Treatments

**Figure 2:**
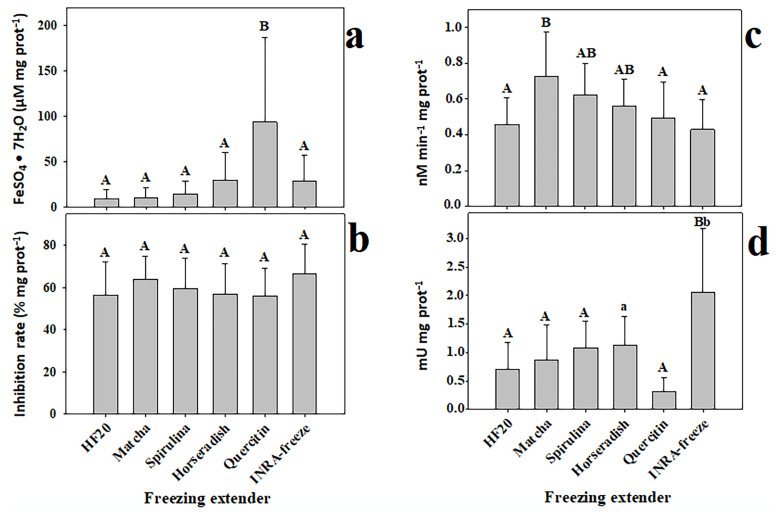
Antioxidant activities in frozen-thawed stallion spermatozoa cryopreserved in HF-20 alone or supplemented with matcha, spirulina, or horseradish extracts or quercetin, as well as in INRA Freeze. (**a**) FRAP; (**b**) SOD activity; (**c**) CAT activity; (**d**) GR activity. Different letters indicate significant differences at *p* < 0.05 (lower case letters) or *p* < 0.01 (capital letters).

Figure 2 shows antioxidant activities detected in sperm cells frozen with the extender supplemented or not (negative control, HF-20) with matcha, spirulina, or horseradish extracts or with quercetin as well as with a commercial freezing extender (INRA Freeze). FRAP activity differed (*p* < 0.001) among treatments, with the highest values found in samples cryopreserved with quercetin (146.75 ± 93.61 µM). CAT activity was significantly influenced by the antioxidant treatments (*p* < 0.001), with the highest values found in the matcha group (0.73 ± 0.25), which significantly (*p* < 0.01) differed from HF-20, Quercetin, and INRA-freeze groups. GR activity did not differ significantly among the experimental treatments. However, samples cryopreserved with INRA-Freeze showed significantly higher GR activity than those cryopreserved with HF-20 alone or HF-20 supplemented with matcha extract, spirulina extract, or quercetin (*p* < 0.01), as well as horseradish extract (*p* < 0.05). SOD activity did not differ among antioxidant-added treatments (*p* = 0.08). The intraclass correlation coefficients (ICC), representing the variable proportion of the total variance attributable to differences among stallions, indicated that the contribution of stallion-related variability differed among the antioxidant parameters, ranging from 0.15 for FRAP to 0.53 for SOD activity, with intermediate values for CAT (0.35) and GR (0.22) activities.

To investigate the potential relationship between the antioxidant activities measured in seminal plasma and sperm lysate from frozen semen samples, either in the presence or absence of natural antioxidant substances, and sperm freezability, stallions were ranked in ascending order according to freezability. Freezability was expressed as the ratio between post-thaw sperm kinetic values and those of the corresponding fresh semen. Progressive motility was selected as the discriminating parameter [32,33], and a threshold value of 35% was used to divide the 10 stallions into two subgroups of five animals each, classified as poor or good freezers. Comparison of the two subgroups, reported in Table S1, showed that, apart from the expected differences in sperm kinetics, no substantial differences in sperm antioxidant activity were detected, except for higher CAT activity in the good-freezer subgroup in both the control and horseradish-treated groups. Unexpectedly, no significant age-related differences were observed between the two groups.

### 3.4. Integrated Multivariate and Regression Analysis of Sperm Quality Traits

**Figure 3:**
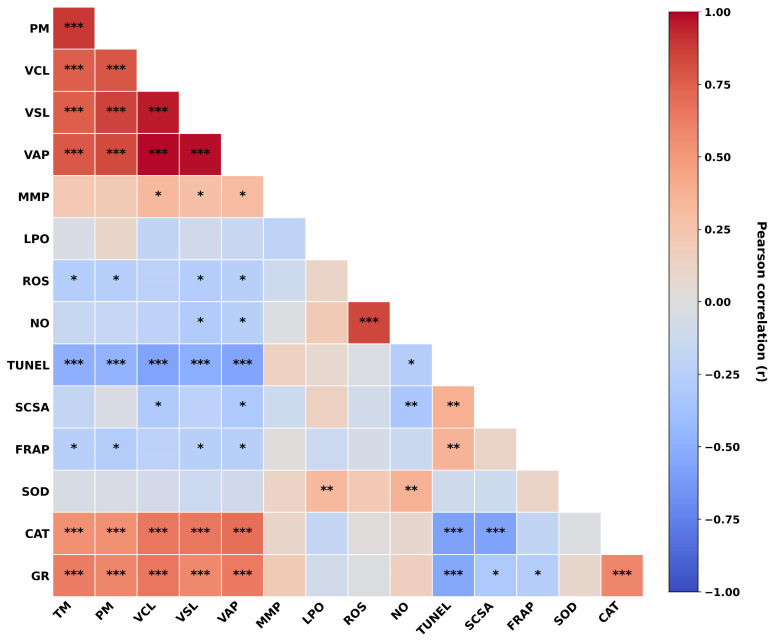
Heatmap of the correlation matrix of sperm kinetics (TM, PM, VAP, VCL, VSL), mitochondrial membrane potential (MMP), OS markers (ROS, LPO, NO), DNA fragmentation indices (TUNEL, SCSA) and antioxidant activities (FRAP, SOD, CAT and GR) in frozen-thawed spermatozoa. * (*p* ≤ 0.05); ** (*p* ≤ 0.01); *** (*p* ≤ 0.001).

The heatmap of the correlation matrix (Figure 3) revealed strong positive correlations (*p* < 0.001) among sperm kinematic parameters (TM, PM, VCL, VSL, and VAP) in frozen-thawed semen. In contrast, sperm kinematic parameters showed negative correlations with DNA fragmentation indices, as TUNEL (*p* < 0.001) and SCSA (*p* < 0.01). A strong positive correlation was also detected between ROS and NO levels (*p* < 0.001). Furthermore, CAT and GR activities were strongly and positively correlated with all sperm kinematic parameters (*p* < 0.001), while showing negative correlations with DNA fragmentation indices. This association was particularly evident in DFI^TUNEL^, which reached the strongest level of statistical significance (*p* < 0.001).

**Figure 4:**
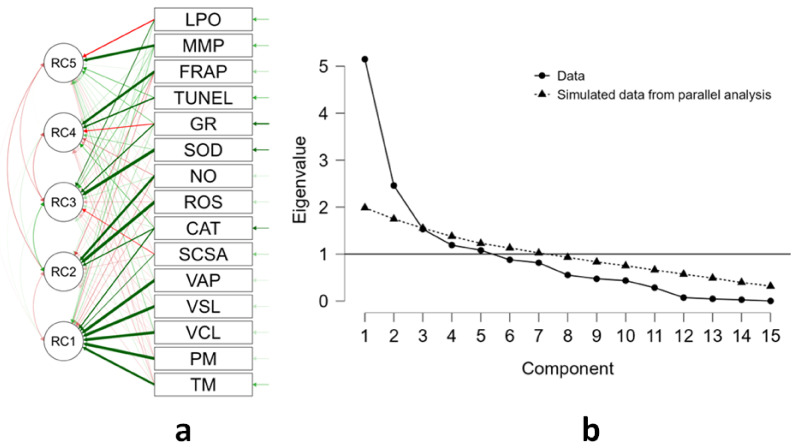
Principal component analysis (PCA) of sperm kinetics (TM, PM, VAP, VCL, VSL), mitochondrial membrane potential (MMP), oxidative/nitrosative stress markers (ROS, LPO, NO), DNA fragmentation indices (TUNEL, SCSA) and antioxidant activities (FRAP, SOD, CAT, and GR activities) in frozen-thawed spermatozoa. (**a**) Path diagram; (**b**) Scree plot.

Principal component analysis (PCA) was performed on standardized variables related to sperm kinetics, bioenergetics, OS, DNA fragmentation and antioxidant activity to reduce dimensionality. Sampling adequacy was acceptable (KMO = 0.606), and Bartlett’s test of sphericity was significant (χ^2^ = 873.568, df = 105, *p* < 0.001), supporting the suitability of the dataset for factor extraction. Based on eigenvalues > 1, scree plot inspection, and parallel analysis, five rotated components (Promax rotation) were retained (Figure 4, Table 3), explaining 73.0% of the total cumulative variance (RC1 = 32.2%, RC2 = 13.2%, RC3 = 11.6%, RC4 = 10.1%, RC5 = 9.0%). The first component was mainly associated with sperm kinematic parameters (VAP, VSL, PM, VCL, TM), while subsequent components captured gradients related to redox balance (CAT, ROS, NO, SOD, GR, FRAP); DNA integrity (TUNEL, SCSA), and mitochondrial and lipid peroxidation markers (MMP, LPO). Component correlations were low to moderate, suggesting a multidimensional structure of the dataset. Overall, the PCA provided an exploratory representation of partially distinct patterns of covariation associated with sperm functional, oxidative, and cellular quality traits.

## 4. Discussion

The freezing sensitivity of stallion semen is strongly affected by inter-individual variability [20], mainly due to differences in intrinsic sperm cryotolerance, among which oxidative stress (OS) is a major contributing factor [6]. Both seminal plasma (SP) and spermatozoa are endowed with a complex antioxidant system that scavenges reactive species and limits oxidative damage [34,35]. However, semen processing for cryopreservation typically involves dilution or removal of SP, leading to a reduction in endogenous antioxidant defences and a consequent increase in susceptibility to redox imbalance [13,36,37].

The present study evaluated enzymatic and non-enzymatic antioxidant activities in SP and sperm cytosol from fresh and cryopreserved semen using the semi-defined extender HF-20, either supplemented or not with natural antioxidant sources, with INRA Freeze serving as a reference. While limited information is available on the effects of matcha and spirulina on sperm cryopreservation in some species, to the best of our knowledge, no studies have specifically evaluated the use of horseradish (*Armoracia rusticana*) in sperm cryopreservation.

The higher enzymatic and non-enzymatic antioxidant activity observed in spermatozoa than in seminal plasma agrees with the findings of Kankofer et al. [38], who reported approximately threefold higher glutathione peroxidase and catalase (CAT) activities and nearly twofold higher superoxide dismutase (SOD) activity in spermatozoa than in seminal plasma immediately after semen dilution. The correlation between SP and sperm cytosol for either SOD or GR activities suggests a degree of coordinated distribution or regulation of these enzymatic antioxidants between the extracellular and intracellular compartments. Conversely, the lack of association for FRAP and CAT may reflect a compartment-specific regulation, supporting the view that the seminal antioxidant network is complex and heterogeneous. However, when these enzymatic activities were correlated with post-thaw sperm parameters, no significant relationships were detected, except for a marginal association between SOD activity in SP and lipid peroxidation (LPO) in frozen-thawed spermatozoa (*p* = 0.057). These findings partially agree with those reported by Papas et al. [23], who proposed SOD activity in SP as a predictor of sperm freezability, based on its positive association with post-thaw motility and viability. Nevertheless, in the present study, the limited strength of the association indicates that this potential predictive role should be interpreted cautiously and requires further validation in larger datasets.

Despite the well-established role of OS in cryodamage, supplementation of freezing extenders with natural antioxidant sources in the present study resulted only in selective modulation of specific antioxidant pathways without producing measurable improvements in kinematic, bioenergetics, oxidative/nitrosative stress, or DNA fragmentation parameters [15]. This apparent discrepancy between biochemical modulations and functional outcomes suggests that the effects of antioxidant supplementation may be complex and context-dependent. Indeed, the efficacy of exogenous antioxidants may depend on the individual baseline redox status. Stallions with lower intrinsic antioxidant capacity may benefit from supplementation, whereas in individuals with adequate endogenous defences, antioxidant addition may yield inconsistent, or even adverse, effects. This interpretation is supported by evidence indicating that stallion identity accounts for a substantial proportion of the variability in post-thaw semen quality [5], although the generally low repeatability of seminal traits should also be considered [1,5]. Furthermore, the absence of a clear distinction between the antioxidant-treated groups and the untreated control may be partly explained by the composition of the HF-20 extender, which includes 10% egg yolk as the only undefined component. Egg yolk, rich in lipids and proteins, plays a protective role in preserving sperm membrane integrity, but it also exhibits considerable antioxidant activity [39,40]. However, replacing egg yolk with alternative components such as soy lecithin is challenging, as soy lecithin itself may exert strong antioxidant effects [41,42]. Conversely, the use of a commercial extender such as INRA Freeze, whose formulation is proprietary for patent reasons, would make it even more difficult to draw robust conclusions regarding the specific effects of antioxidant supplementation.

Consistent with this framework, the analysis of antioxidant markers in the present study further suggests a possible contribution of inter- and intra-individual variability. SOD activity showed a strong stallion-specific component (ICC = 0.53), suggesting that it may partially reflect intrinsic characteristics. In contrast, FRAP (ICC = 0.15) and GR (ICC = 0.22) showed greater intra-individual variability, which may be related to the influence of extrinsic factors such as treatment and extender composition. CAT exhibited an intermediate pattern (ICC = 0.35), suggesting that both intrinsic and extrinsic factors may contribute to its variability. Altogether, these results indicate that extender composition and individual biological variability may both influence the antioxidant response of spermatozoa, and should, therefore, be carefully considered when developing and optimizing cryopreservation strategies.

The possible functional relevance of antioxidant defences is also suggested by regression analysis, which indicated associations between CAT and GR activities and sperm kinetics after thawing. Specifically, both enzymes were positively correlated with kinematic parameters and negatively associated with DNA fragmentation indices, suggesting a potential involvement in the cryopreservation-induced damage. Accordingly, CAT and GR may represent sensitive intracellular indicators associated with post-thaw sperm functionality. These findings are in agreement with previous studies reporting relationships between antioxidant systems and sperm quality. For instance, CAT activity is reduced in the SP of infertile men compared with normozoospermic individuals [43]. In stallions, SOD, PON1, and TEAC-based total antioxidant capacity (TAC) in SP are positively associated with post-thaw sperm motility and membrane integrity [13]. Moreover, exogenous antioxidant supplementation, including SOD, CAT, and GPx, has been shown to improve stallion sperm viability, motility, and DNA integrity during cooled storage [44], with comparable protective effects also reported in other species, including rams [45].

Although numerous studies have focused on semen extender supplementation with purified natural antioxidant compounds such as quercetin, more recent research has focused on plant-derived extracts, including matcha and spirulina, for cryopreservation applications [27,29,30,46,47,48]. The use of horseradish extract, as a potential additive to sperm freezing extenders, represents an entirely novel approach, although evidence exists from studies conducted with extracts from another species, *Moringa oleifera*, which is sometimes mistakenly associated with horseradish despite having different nutraceutical properties [49,50]. However, the results reported so far remain inconsistent, and the effects of these extracts on intracellular antioxidant systems are still largely unexplored. In stallions, spirulina supplementation has been associated with increased SOD and GR activities, as well as TAC, together with improved sperm motility and reduced lipid peroxidation [27]. Similarly, in buffalo semen, spirulina extract has been reported to increase SOD and GSH activities and TEAC values in post-thaw sperm [46]. Green tea (*Camellia sinensis*) extracts, which differ from matcha in terms of cultivation and processing methods, have also been shown to improve several sperm quality parameters in frozen-thawed semen, while also increasing total antioxidant capacity (TAC) [30] and glutathione levels [51]. Nevertheless, their effects on specific enzymatic antioxidants, such as SOD and glutathione peroxidase (GPx), remain inconsistent [30].

The absence of clear functional improvements observed in stallion sperm cryopreserved with these antioxidant compounds, despite the modulation of antioxidant activities, suggests that different antioxidant sources may not exert equivalent biological effects. Their efficacy may depend on factors such as bioavailability, mechanism of action, dosage, and interactions with sperm physiology.

To obtain a broader exploratory overview of the effects of these antioxidant treatments, the values of enzymatic activities and total antioxidant capacity (TAC) in seminal plasma (SP) and sperm cytosol were integrated with previously analyzed variables related to sperm kinetics, OS, mitochondrial activity, and DNA integrity [15]. The resulting dataset was then subjected to multivariate analysis using principal component analysis (PCA) with the aim of exploring patterns of covariation among variables, reducing dimensionality, and identifying potential latent structures within the dataset. However, given the limited sample size and the relatively large number of variables included, this analysis should be considered exploratory and hypothesis-generating rather than providing solid and definitive findings.

The first rotated component (RC1) of PCA was mainly defined by sperm kinematic variables (VAP, VSL, VCL, PM, and TM), suggesting a coherent motility-related dimension with the known interdependence among CASA-derived parameters [52]. CAT showed a moderate cross-loading on RC1 and RC2, suggesting a possible association between antioxidant defence and sperm motility, in agreement with regression results and previous studies linking antioxidant enzymes and sperm function [53,54,55]. However, this should not be interpreted as a direct causal relationship. Supporting this interpretation, supplementation of the extender with a CAT inhibitor has been shown to impair sperm motility and increase intracellular H_2_O_2_ levels in stallion spermatozoa [56]. The second component (RC2) was mainly associated with oxidative and nitrosative stress markers (ROS and NO), likely reflecting a redox imbalance-related axis. Its separation from RC1 suggests that oxidative status and motility represent partially distinct, although interacting, aspects of sperm physiology [57,58,59]. This pattern may explain why biochemical modulation of redox variables does not always result in major sperm functional improvements. More broadly, these findings support evidence implicating oxidative and nitrosative stress in cryopreservation-induced sperm damage [55,56,57]. The third component (RC3), mainly driven by SOD with a contribution from GR, showed an inverse association with DNA damage, suggesting that antioxidant treatments enhancing enzymatic defences may support chromatin protection [53,60]. The dual loading of GR, including its negative contribution to RC4, may reflect the context-dependent role of this enzyme in redox regulation. In contrast, the weaker associations observed for non-enzymatic FRAP-based antioxidant capacity suggest that the mere presence of antioxidant compounds may be insufficient to prevent oxidative damage, particularly at the DNA level. The fourth component (RC4) was mainly associated with the FRAP-based TAC and DFI^TUNEL^ (positive loadings), with a small negative contribution from GR. Rather than indicating a direct protective relationship, this pattern suggests that both variables respond in parallel to common factors related to redox imbalance during cryopreservation. According to Catalán et al. [13], FRAP-based TAC is not a reliable marker for discriminating between good and poor stallion sperm freezers, unlike enzymatic (PON1, SOD) and non-enzymatic (TEAC-based TAC) markers, which are more strongly associated with stallion sperm cryotolerance. Therefore, this component should be interpreted cautiously. Finally, RC5 was largely defined by MMP, suggesting that mitochondrial functionality represents a distinct domain within the dataset. This may indicate that mitochondrial function is not fully explained by the antioxidant variables examined and may not be completely preserved by general antioxidant supplementation. In post-thaw stallion spermatozoa, Catalán et al. [13] found no significant correlation between MMP and most SP antioxidant activities except GPx.

Overall, the PCA provides a useful exploratory framework to describe how sperm kinetics, oxidative status, antioxidant defenses, DNA integrity, and mitochondrial function covaried within the present dataset. The results are consistent with the view that these parameters may be organized into interconnected yet partially distinct functional domains, which may respond differentially to cryopreservation and antioxidant supplementation. However, because of the limited sample size and the high number of variables included in the analysis, these findings should be regarded as supportive and hypothesis-generating. They do not provide strong biological evidence on their own, but they may help to guide future studies designed to test specific mechanistic relationships. In this context, the present exploratory results suggest that antioxidant supplementation may modulate selected redox-related and motility-associated variables, whereas its effects on DNA integrity and mitochondrial function may be limited, indirect, or dependent on specific mechanisms. Further studies with larger sample sizes and targeted experimental designs are needed to confirm these patterns and to develop mechanism-based supplementation strategies for improving sperm cryotolerance.

## 5. Conclusions

This study shows that the addition of certain natural extracts with antioxidant activity to the sperm cryopreservation extender, despite inconsistent results regarding post-thaw sperm functionality, produces antioxidant-dependent effects capable of modulating some antioxidant activities within sperm cells. This could be useful in cases of prolonged sperm manipulation, such as sperm sex-sorting. The differences in antioxidant potential observed among antioxidant treatments with natural products indicate that their specific composition may influence the redox balance of sperm cells after thawing. The associations detected between antioxidant activities and sperm functional parameters are consistent with the role of oxidative homeostasis in post-thaw sperm quality, although these relationships should be interpreted cautiously and do not imply direct causality. Notably, this study provides an integrated evaluation of antioxidant activities in sperm lysates together with sperm kinetics, bioenergetics, oxidative/nitrosative stress markers, and DNA integrity, offering an exploratory overview of the relationships between redox balance and sperm function after cryopreservation. Nevertheless, further studies with larger sample sizes and targeted experimental designs are required to confirm these findings, elucidate the underlying mechanisms, and optimize extender formulations for practical application in breeding programs.

## Figures and Tables

**Table 1:** Antioxidant activities (mean ± SD) in seminal plasma and sperm lysates from fresh samples. Data are normalized to the protein content of each sample (mg^−1^ protein).

	Seminal Plasma	Sperm Lysates
FRAP (µM FeSO_4_·7H_2_O)	25.80 ± 10.24	48.76 ± 9.59
SOD (inhibition rate, %)	10.43 ± 4.76	58.88 ± 9.28
Catalase (nM min^−1^ mL^−1^)	0.46 ± 0.22	2.55 ± 0.92
Glutathione reductase (mU mL^−1^)	0.82 ± 0.57	4.37 ± 2.19

**Table 2:** Kinematic, bioenergetics, oxidative/nitrosative stress and DNA fragmentation parameters in stallion sperm, either fresh (T0) or cryopreserved in HF-20 extender alone or supplemented with natural extracts (matcha, spirulina, or horseradish) or quercetin or INRA Freeze.

	T0	HF-20	Matcha	Spirulina	Horseradish	Quercetin	INRA Freeze
	Mean ± SD	Mean ± SD	Mean ± SD	Mean ± SD	Mean ± SD	Mean ± SD	Mean ± SD
TM	85.5 ± 15.1 ^A^	46.8 ± 22.1 ^B^	48.0 ± 21.1 ^B^	48.3 ± 18.3 ^B^	43.2 ± 17.9 ^B^	42.1 ± 19.4 ^B^	47.7 ± 21.7 ^B^
PM	37.0 ± 12.9 ^A^	12.8 ± 10.7 ^B^	15.9 ± 13.6 ^B^	17.8 ± 13.1 ^B^	12.1 ± 11.8 ^B^	12.5 ± 13.8 ^B^	11.7 ± 9.5 ^B^
VCL	89.4 ± 14.6 ^A^	36.2 ± 15.8 ^B^	37.6 ± 15.2 ^B^	42.1 ± 13.8 ^B^	37.6 ± 15.0 ^B^	36.1 ± 16.8 ^B^	35.3 ± 12.7 ^B^
VSL	36.0 ± 4.8 ^A^	16.5 ± 7.3 ^B^	17.6 ± 8.0 ^B^	20.4 ± 7.6 ^B^	17.3 ± 7.0 ^B^	16.3 ± 7.8 ^B^	15.6 ± 5.4 ^B^
VAP	48.1 ± 6.7 ^A^	20.6 ± 8.4 ^B^	21.5 ± 8.8 ^B^	24.4 ± 8.0 ^B^	21.4 ± 8.0 ^B^	20.1 ± 9.0 ^B^	19.8 ± 6.7 ^B^
MMP	8.1 ± 3.7	6.8 ± 5.4	8.4 ± 8.4	5.8 ± 3.6	7.5 ± 5.4	4.0 ± 2.3	6.5 ± 6.0
LPO	12.7 ± 3.5	16.0 ± 4.9	17.9 ± 8.7	20.5 ± 10.5	19.1 ± 9.5	15.5 ± 5.4	17.3 ± 6.2
ROS content	2.7 ± 1.1	2.0 ± 0.7	4.7 ± 4.9	3.0 ± 2.0	2.4 ± 1.1	2.8 ± 1.5	2.8 ± 0.8
NO content	3.0 ± 0.6	2.1 ± 1.0	3.8 ± 3.1	2.4 ± 1.3	2.2 ± 1.1	2.4 ± 1.5	3.2 ± 1.3
DFI^TUNEL^	4.4 ± 0.6 ^A^	6.1 ± 0.5 ^B^	5.9 ± 0.5 ^B^	6.3 ± 0.4 ^B^	6.2 ± 0.1 ^B^	6.2 ± 0.4 ^B^	5.6 ± 1.0 ^B^
DFI^SCSA^	6.2 ± 0.8 ^A^	6.8 ± 0.4 ^B^	7.0 ± 0.2 ^B^	6.9 ± 0.2 ^B^	6.9 ± 0.2 ^B^	7.0 ± 0.2 ^B^	6.9 ± 0.5 ^B^

Total motility (TM, %), progressive motility (PM, %), curvilinear velocity (VCL, µm s^−1^), straight-line velocity (VSL, µm s^−1^), average path velocity (VAP, µm s^−1^), mitochondrial membrane potential (MMP), lipid peroxidation (LPO), reactive oxygen species (ROS), nitric oxide (NO), DNA fragmentation index determined by the TUNEL (DFI^TUNEL^) and SCSA (DFI^SCSA^) assays. MMP was calculated as the ratio of the second (~595 nm) to the first (~535 nm) fluorescence intensity peak (J0B/J0A). LPO was calculated as the ratio of the first (~520 nm) fluorescence intensity peak to the sum of the first (~520 nm) and second (~590 nm) fluorescence intensity peaks, expressed as C0A/(C0A + C0B). Fluorescence intensity is reported in arbitrary units (a.u.). DFI^TUNEL^ was calculated as the ratio of the green emission peak to the red emission peak. DFI^SCSA^ was calculated as the ratio of the red emission peak (647 nm, F0R) to the sum of the red (647 nm, F0R) and the green (~530 nm, F0G) emission peaks. ^A^, ^B^ (*p* < 0.01).

**Table 3:** Component loadings from principal component analysis (PCA) of sperm kinetics, mitochondrial membrane potential, oxidative/nitrosative stress markers, DNA fragmentation indices and antioxidant activities in frozen-thawed spermatozoa (Promax rotation was applied).

	RC1	RC2	RC3	RC4	RC5	Uniqueness
VAP	0.933					0.077
VSL	0.932					0.097
PM	0.926					0.086
VCL	0.902					0.097
TM	0.783					0.301
CAT	0.542	0.531				0.274
SCSA	0.433		−0.418			0.597
ROS		0.961				0.107
NO		0.817				0.080
SOD			0.957			0.169
GR			0.542	−0.470		0.296
LPO			0.445		−0.530	0.407
FRAP				0.841		0.330
TUNEL				0.605		0.459
MMP					0.858	0.206

## Data Availability

The original contributions presented in this study are included in the article. Further inquiries can be directed to the corresponding authors.

## Supplementary Materials

The following supporting information can be downloaded at: https://www.mdpi.com/article/10.3390/ani16111704/s1, Table S1. Antioxidant activity and sperm freezability.
