# Supplementary material for: Antioxidant Activity of Stallion Spermatozoa After Cryopreservation with Natural Antioxidant-Supplemented Extenders
Source: Animals (Basel). 2026 Jun 2;16(11):1704. doi: 10.3390/ani16111704 (PMC13256018; doi:10.3390/ani16111704)
Supplement: Supplementary file 1 [file animals-16-01704-s001.zip › animals-4307554-supplementary.pdf]

Supplemented materials

Antioxidant Activity of Stallion Spermatozoa after Cryopreservation with Natural Antioxidant-Supplemented Extenders

Stefano Cecchini Gualandi, Alessandro Pistone, Angela Ostuni, Graziano Preziosi, Maria Antonietta Ferrara and Raffaele Boni

Table S1. **Antioxidant activity and sperm freezability.** Antioxidant activity, assessed by ferric reducing antioxidant power (FRAP), superoxide dismutase (SOD), catalase (CAT), and glutathione reductase (GR), was measured in seminal plasma (SP) and sperm lysates from spermatozoa cryopreserved in a freezing extender (HF-20) supplemented either without antioxidants (C0) or with selected antioxidant compounds, including matcha (M), spirulina (S), horseradish (R) extracts, or quercetin (Q), as well as in a commercial extender (INRA Freeze). Stallions were stratified according to semen freezability, defined as the ratio of frozen-thawed to fresh kinematic parameters, and classified as good or poor freezers by dividing the animals into two equal-sized subgroups based on progressive motility (PM threshold = 35%). Freezability was further evaluated using total motility (TM), progressive motility (PM), curvilinear velocity (VCL), straight-line velocity (VSL), and average path velocity (VAP).

		**Poor freezers** Mean ± SD	**Good freezers** Mean ± SD	**p value**
Stallions	n.	5	5	
AGE	years	10.4 ± 5.1	9.2 ± 6.3	0.7487
TM	%	35.8 ± 16.9	78.8 ± 23.2	0.0102**
PM	%	15.5 ± 10.5	49.8 ± 9.6	0.0007**
VCL	%	30.4 ± 10.6	50.3 ± 16.7	0.0551
VSL	%	34.7 ± 12.8	55.3 ± 16.2	0.0563
VAP	%	32.4 ± 10.6	52.9 ± 16.3	0.0456*
FRAP_SP	%	27.5 ± 9.3	24.1 ± 11.9	0.6334
SOD_SP	%	7.8 ± 1.8	13.1 ± 5.5	0.0717
CAT_SP	%	0.3 ± 0.1	0.6 ± 0.3	0.1155
GP_SP	%	0.7 ± 0.5	0.9 ± 0.7	0.5244
FRAP_C0	%	61.9 ± 7.6	68.7 ± 10.5	0.2717
SOD_C0	%	57.9 ± 17.0	54.9 ± 16.7	0.7814
CAT_C0	%	0.3 ± 0.1	0.6 ± 0.1	0.0041*
GP_C0	%	0.7 ± 0.5	0.7 ± 0.5	0.8339
FRAP_M	%	58.6 ± 12.1	67.3 ± 7.2	0.2034
SOD_M	%	63.0 ± 12.2	65.2 ± 10.2	0.7683
CAT_M	%	0.7 ± 0.3	0.8 ± 0.2	0.6563
GP_M	%	0.9 ± 0.6	0.9 ± 0.7	0.9907
FRAP_S	%	54.4 ± 9.6	61.2 ± 18.0	0.4785
SOD_S	%	65.0 ± 14.3	54.3 ± 13.5	0.2597
CAT_S	%	0.6 ± 0.2	0.7 ± 0.2	0.219
GP_S	%	0.9 ± 0.5	1.3 ± 0.4	0.2114
FRAP_R	%	95.7 ± 30.0	77.8 ± 30.0	0.3745
SOD_R	%	61.4 ± 17.5	52.4 ± 11.1	0.3622
CAT_R	%	0.5 ± 0.1	0.7 ± 0.1	0.0353*
GP_R	%	1.1 ± 0.5	1.2 ± 0.6	0.8182
FRAP_Q	%	151.0 ± 106.5	142.5 ± 91.2	0.8962
SOD_Q	%	56.7 ± 16.8	55.7 ± 10.2	0.9052
CAT_Q	%	0.4 ± 0.1	0.6 ± 0.2	0.09
GP_Q	%	0.3 ± 0.2	0.3 ± 0.3	0.9774
FRAP_INRA Freeze	%	49.0 ± 6.4	76.2 ± 36.6	0.1397
SOD_INRA Freeze	%	63.6 ± 20.1	69.4 ± 3.6	0.5404
CAT_INRA Freeze	%	0.3 ± 0.1	0.5 ± 0.2	0.1101
GP_INRA Freeze	%	1.6 ± 0.4	2.5 ± 1.5	0.2196

* ($p < 0.05$); ** ($p < 0.01$)
